# Evaluation and modification of tumor cell isolation techniques from malignant effusions for rapid drug sensitivity testing

**DOI:** 10.1002/1878-0261.70072

**Published:** 2025-06-17

**Authors:** Navit Mooshayef, Hana Barhom, Sumit Chatterji, Netta Hecht, Oran Warhaftig, Iris Kamer, Oranit Zadok, Jair Bar, Limor Broday, Amir Onn, Michael Peled

**Affiliations:** ^1^ Institute of Pulmonary Medicine Chaim Sheba Medical Center Ramat Gan Israel; ^2^ Faculty of Medical & Health Sciences Tel Aviv University Israel; ^3^ Institute of Oncology Chaim Sheba Medical Center Ramat Gan Israel; ^4^ Department of Cell and Developmental Biology, Faculty of Medical & Health Sciences Tel Aviv University Israel

**Keywords:** CD45, drug sensitivity testing, EpCAM, lung cancer, pleural effusion, tumor cell isolation

## Abstract

Non‐small cell lung cancer treatment decisions rely on several diagnostic steps. Tests that rely on DNA sequencing often fail to capture the full mutational landscape of tumor cells, and drug sensitivity testing (DST) has limitations hindering widespread use currently. One of the major challenges for DST is the rapid isolation of a sufficient number of live tumor cells that would allow testing of multiple drugs simultaneously. To address this challenge, we have developed a DST procedure specifically tailored for tumor cells originating from malignant pleural effusions. We first identified tumor cells by anti‐epithelial cell adhesion molecule (EpCAM) flow cytometry and then compared several methods for tumor cell isolation: immunomagnetic enrichment of epithelial cells using EpCAM, negative selection via immunomagnetic CD45^+^ cell depletion, and size‐based separation and capture of tumor cells utilizing cell strainers. Of these methods, repeated rounds of CD45^+^ cell depletion, in which the number of rounds is set by the initial percentage of tumor cells in the sample, were the most effective. By combining tumor cell enrichment with DST, we have developed a system which generates DST results that correlate with clinical outcomes.

## Graphical abstract

Non‐small cell lung cancer targeted treatment is limited to a few known genetic alterations, with few alternatives in advanced treatment lines. To direct treatment decisions by drug sensitivity testing (DST), this study compared several methods for tumor cell isolation from malignant effusions, pointing to repeated CD45^+^ cell depletion for effective tumor cell isolation, yielding DST results that correlated with clinical outcomes.

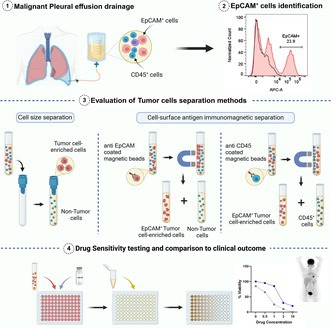

**Abbreviations**
ALK: anaplastic lymphoma kinase
BSA: bovine serum albumin
CT: computed tomography
CTC: circulating tumor cells
DMSO: dimethyl sulfoxide
DSS: drug sensitivity score
DST: drug sensitivity testing
EDTA: ethylenediaminetetraacetic acid
EGFR: epidermal growth factor receptor
EMT: epithelial‐to‐mesenchymal transition
FACS: fluorescence‐activated cell sorting
FBS: fetal bovine serum
FcR: Fc receptor
FFPE: formalin‐fixed paraffin‐embedded tissue
FSC‐SSC: forward scatter‐side scatter
H&E: hematoxylin and eosin
IHC: immunohistochemistry
MPE: malignant pleural effusion
NGS: next‐generation sequencing
NSCLC: non‐small cell lung cancer
PBS: phosphate‐buffered saline
PCR: polymerase chain reaction
PE: pleural effusion
PET: positron emission tomography
RBC: red blood cells
RT: room temperature
TKI: tyrosine kinase inhibitor
VAF: variant allele frequency

## 1 Introduction

Advancements in DNA sequencing have facilitated the classification of approximately 30% of non‐small cell lung cancer (NSCLC) patients based on specific tumor driver genetic alterations [1, 2]. Over the past two decades, small molecules, primarily tyrosine kinase inhibitors (TKIs), targeting mutations or translocations in ALK, ROS, NTRK, MET, EGFR, KRAS, and BRAF have been clinically approved for NSCLC patients, resulting in complete or partial responses in most cases [3, 4, 5].

However, most patients eventually succumb to disease progression due to treatment resistance, driven by point mutations of the kinase domain, other kinase gene amplification, and activation of compensatory pathways. These resistance mechanisms may respond to different TKIs other than those indicated for the primary mutation [6, 7].

Moreover, current genetic testing, used for determining targeted treatment for cancer patients, provides only a partial analysis of the mutational landscape, especially in advanced‐stage cancer patients [8, 9]. Thus, an unbiased drug screen that includes all the available targeted therapies is necessary to rapidly identify the best next‐line treatment for these patients.

Drug screens, also known as drug sensitivity testing (DST) assays, assess the viability of tumor cells following exposure to different drugs in order to determine the drug that effectively reduces tumor cell viability/proliferation at the lowest dose [5, 10].

Despite years of research in this subfield of personalized medicine, no DST assay is currently translated into routine clinical management of patients due to limited clinical evidence linking DST results to therapeutic outcomes [11]. The historical lack of correlation between DST results and clinical outcomes may partially stem from the fact that DST before the emergence of targeted therapies was mainly performed with chemotherapies, which impact a wide range of organs and cells in the body that are not recapitulated *in vitro*. However, with the appearance of targeted therapies, which minimally impact nontumor cells, *in vitro* tumor DST results may show increased correlation with clinical outcomes.

Another limitation of historical DST assays is the extended time between the biopsy and the results of the drug screen, as many DST platforms rely on the establishment of tumor organoids or primary cancer cell lines [5, 12], which can take up to 20 weeks, while advanced stage cancer patients require immediate treatment.

In addition, and even before obtaining DST results, it is necessary to rapidly identify tumor cells in the sample. Pathological assessment of solid biopsies and cytology of body fluids are currently the gold standard for cancer diagnosis; however, results are usually received not sooner than 2–7 days following tissue procurement and require assessment by a pathologist [13]. To establish rapid DST, which relies on the presence of live, fresh tumor cells, a quicker identification process is essential.

Here, we provide a practical and fast method for cancer DST, based on targeted drugs, that can deliver a recommendation for personalized cancer treatment in 4 days. For this purpose, we utilized malignant pleural effusions (MPEs), which accumulate in more than 20% of lung cancer patients [14, 15], as a source of tumor cells. To rapidly identify tumor cells in MPEs, we employed flow cytometry identification of epithelial cell adhesion molecule (EpCAM), a marker for tumor cells in MPE [16, 17]. Next, since MPEs are heterogeneous and tumor cells represent only a fraction of the total MPE cell population [18], tumor cell isolation is required before testing their drug sensitivity. Thus, we compared several tumor cell isolation methods to formulate an efficient tumor cell isolation procedure for enriching MPE‐derived tumor cells that are adequate for drug screening.

## 2 Materials and methods

### 2.1 Experimental model and study participant details

#### 2.1.1 Pleural effusion collection and cell purification

This study involves human participants, conforms to the standards set by the Declaration of Helsinki, and was approved by the local ethics committee, SMC‐5494‐18. The experiments were undertaken with the understanding and written consent of each subject.

Pleural fluid samples from 60 patients with various diseases (Table S1) were collected at Chaim Sheba Medical Center during September 2021 to January 2024. To prevent clotting and clumping, sterile EDTA was added immediately (final concentration 10 mm). Sample centrifugation (20 min, 800 ***g***) separated the cells from the fluid. Next, RBC lysis buffer (BioLegend, San Diego, CA, USA; 420301) was used to eliminate red blood cells, followed by another centrifugation (5 min, 500 ***g***). Finally, the cells were filtered through a 70‐μm mesh and frozen in 90% FBS and 10% DMSO.

#### 2.1.2 Pleural effusion samples—clinical data and cytology

Data from the patients included the following: age at evaluation, gender, tumor history, diagnostic imaging (chest radiography, computerized tomography and positron emission tomography), cytopathology and/or histopathology data. Cytopathological analysis was performed by conventional microscopy, and findings were reported according to the international system for reporting serous fluid cytology [19]. All pleural effusion (PE) samples were examined using smears. Immunohistochemistry (IHC) of cell blocks was only performed for samples with a high percentage of malignant cells. MPE was established when tumor cells were detected in pleural effusion by cytology.

### 2.2 Methods details

#### 2.2.1 EpCAM immunohistochemistry

MPE purified cells were collected in Eppendorf tubes and centrifuged at 400 ***g*** for 5 min; the pellet was washed with cold PBS and fixed with 1 : 1 96% ethanol: 4% paraformaldehyde for 5 min. The fixed pellet was centrifuged at 1000 ***g*** for 5 min and resuspended in 5–20 μL melted Bio‐Agar gel (Bio‐Optica, Milano, Italy). The gel containing the cells was flattened to a thin disk, cooled at ~ 0 °C, coated on both sides with additional melted Bio‐Agar gel, cooled again, followed by routine FFPE processing. Briefly, the tissues were dehydrated using a series of increasing concentrations of alcohol (70%, 80%, 90%, 95%, and 100%) and then removal of the dehydrant with xylene. Finally, the tissue was infiltrated with paraffin and manually placed into a block. Tissues embedded in paraffin were sectioned into 3.5 μm slices and mounted on microscope slides. The slides were placed in an oven for 1 h at 60 °C before proceeding to the staining steps. H&E stains were performed automatically on a ST5020 device (Leica Biosystems, Wetzlar, Germany) according to the manufacturer's instructions. Immunohistochemistry stains were performed with EpCAM antibody (1 : 50, M0804, Dako, Glostrup, Denmark) on a Benchmark XT staining module (Ventana Medical Systems., Oro Valley, AZ, USA) using iVIEW DAB Detection Kit (catalog #: 760‐091, Ventana Medical Systems). After immunostaining, sections were counterstained with hematoxylin (Ventana Medical Systems), rinsed in distilled water, and finally dehydrated manually in graded ethanol (70%, 96%, and 100%). Then, the sections were cleared in xylene and mounted with Entellan (Surgipath, Eagle River, WI, USA). IHC images were obtained using Olympus BX50 microscope (Olympus Corporation, Tokyo, Japan).

#### 2.2.2 Flow cytometry

Pleural effusion purified cells were suspended in FACS buffer (2 mm EDTA, 1% BSA in PBS) and blocked with TruStain FcX™ reagent (BioLegend, 422302). Next, the cells were stained with fluorescently conjugated antibodies against EpCAM (9C4, BioLegend, 324208) and CD45 (HI30, BioLegend, 304008). Some cell samples were stained with antibodies against EGFR (AY13, BioLegend, 352907), N‐Cadherin (8C11, BioLegend, 350805), and with Helix NP™ reagent (BioLegend, 425303) in order to evaluate their viability. Events were recorded using CytoFlex (Beckman Coulter, Brea, CA, USA) and analyzed using flowjo software (Ashland, OR, USA).

#### 2.2.3 MPE cell separation by cell size

Pleural effusion cells were suspended in warm RPMI 1640 medium. Samples of an equal number of cells were filtered through pluriStrainer® filters with a mesh size of either 5, 10, or 20 μm (PluriSelect, 43‐50005‐13, 43‐50010‐03, 43‐50020‐03). Both fractions, cells that passed through each strainer and cells that were recovered from the sieve, were collected. The strained fraction and the initial sample, prior to straining, were analyzed by flow cytometry for the evaluation of EpCAM‐positive cells.

#### 2.2.4 EpCAM ^+^ cell isolation

Following RBC lysis, 1 × 10^7^ pleural effusion cells were suspended in plain RPMI 1640 medium and incubated for 30 min at RT with DNase (New England Biolabs, Ipswich, MA, USA; M0303S) at a concentration of 20 IU·mL^−1^ to reduce cell clumping. The cells were then centrifuged and resuspended in Isolation buffer (PBS pH 7.2, 0.5% BSA, 2 mm EDTA). Isolation of EpCAM‐positive cells was performed using MACS cell separation system (Miltenyi Biotec, Bergisch Gladbach, Germany) according to the manufacturer's instructions. Briefly, the cells were blocked using an anti‐FcR reagent (Miltenyi Biotec, 130‐059‐901) and then incubated at 4 °C with 100 μL of CD326 MicroBeads (Miltenyi Biotec, 130‐061‐101) for 30 min. Following an Isolation buffer wash of unbound beads, the magnetically labeled cells were then separated by passing them through MS columns (Miltenyi Biotec, 130‐042‐201) attached to a magnet (Miltenyi Biotec, MiniMACS™ Separator 130‐042‐102, MACS® MultiStand 130‐042‐303). Finally, the column was removed from the magnet and labeled cells were eluted into 3 mL of isolation buffer using a plunger.

#### 2.2.5 CD45 ^+^ cell depletion

Following RBC lysis, 1 × 10^7^ pleural effusion cells were suspended in plain RPMI 1640 medium and incubated for 30 min at RT with DNase (New England Biolabs, M0303S) at the concentration of 20 IU·mL^−1^ to reduce cell clumping. The cells were then centrifuged and resuspended in Isolation buffer (PBS pH 7.2, 0.5% BSA, 2 mm EDTA). Depletion of CD45‐positive cells was performed using MojoSort™ Human CD45 Nanobeads (BioLegend, 480029) and MojoSort™ Magnet 5 mL (BioLegend, 480019) according to the manufacturers' instructions. Briefly, the cells were incubated at 4 °C with 10 μL of MojoSort™ human CD45 Nanobeads for 15 min. Following an Isolation buffer wash of unbound beads, the cell sample was placed in the magnet for 6 min. The labeled cells were retained to the magnet and the unlabeled cell fraction was collected.

#### 2.2.6 KRAS and EGFR mutation analysis

Snap‐frozen cell pellets of MPE cells (‘Input’ cells) and CD45^−^ cells (enriched tumor cells) were utilized to detect KRAS mutation and EGFR deletion allele frequencies. The DNA was extracted using Grisp Genomic DNA Kit (GRISP REASERCH SOLUTIONS, Porto, Portugal; GK02.0100). The desired amplicons were enriched in the first PCR using designated primers with SP1/SP2 tails and labeled with sequencing barcodes in the second PCR. All samples were sequenced using a G400 sequencer in 150PE DNBSEQ Technology (MGI Tech Co., Shenzhen, Guangdong, China).

#### 2.2.7 Drugs

The following drugs were used in the study: osimertinib (Selleck Chemicals, Houston, TX, USA; S7297), afatinib (Selleck Chemicals, S1011), alectinib (Selleck Chemicals, S2762), and lorlatinib (Selleck Chemicals, S7536). Stock solutions were prepared using DMSO, further diluted in complete RPMI medium to the appropriate concentration, and plated in 96‐well plates. The drugs were screened at five concentrations (0.03–3 μm) with matched DMSO concentration vehicle controls.

#### 2.2.8 Drug sensitivity assay

Tumor cells purified from MPE as described above (subsection 2.2.5) were seeded at a density of 1 × 10^4^ cells per well of a 96‐well plate, in 100 μL culture medium (RPMI supplemented with 10% FBS and 1% Pen‐Strep solution). The cells were then immediately treated with the various drugs at the indicated concentrations, and following 72 h of incubation (37 °C, 5% CO_2_ incubator), cell viability was measured using the MTS cell proliferation kit (Abcam, Cambridge, UK; ab197010) according to the manufacturer's instructions. Briefly, 10 μL of fresh MTS tetrazolium solution was added to each well, and the cells were incubated for 3–6 h in an incubator. The reduction in the MTS compound, which serves as an indicator for cellular viability, was measured using the TECAN Spark® microplate reader at a wavelength of 490 nm.

### 2.3 Quantification and statistical analysis

#### 2.3.1 Statistical analyses

Statistical analysis and all the graphs presented here, including dose–response curve fits, were generated using graphpad prism 8 (GraphPad Software Inc, Boston, MA, USA).

A two‐tailed Mann–Whitney test was used to compare the median percentage of EpCAM^+^ cells, determined by flow cytometry, between patients' samples with positive cytological results and patients with negative results.

Three enrichment experiments were performed in order to compare cell recovery and tumor cell enrichment between the described methods.

Percent cell recovery is calculated by the following equation:

$$\;\%\;\text{cell recovery rate}=\begin{array}{l} \frac{\text{Number of live EpCAM positive cells following enrichment}}{\text{Number of initial live EpCAM positive cells}}\\ \times 100 \end{array}$$

Tumor cell enrichment is calculated by the following equation:

$$\;\begin{array}{l} \%\text{EpCAM positive cells fold change}\\ \;=\frac{\%\text{EpCAM positive cells following enrichment}}{\text{initial}\;\%\text{EpCAM positive cells}} \end{array}$$

To assess statistical significance, Welch's ANOVA test or Ordinary one‐way ANOVA test was used.

DST dose–response curves were plotted via the bioinformatic ‘Breeze’ pipeline [20]. Next, dose–response curve parameters were employed to calculate the drug sensitivity score (DSS), as described [21]. Finally, statistical significance of DSS was evaluated using unpaired *t*‐tests.

For all statistical analysis, the results were considered statistically significant if a *P* value < 0.05 was observed. Error bars indicate standard error of the mean. Statistical details and number of samples used for a particular result can be found in the figure legends.

## 3 Results

### 3.1 Rapid and sensitive MPE diagnosis using anti‐EpCAM FACS analysis

**Fig. 1:**
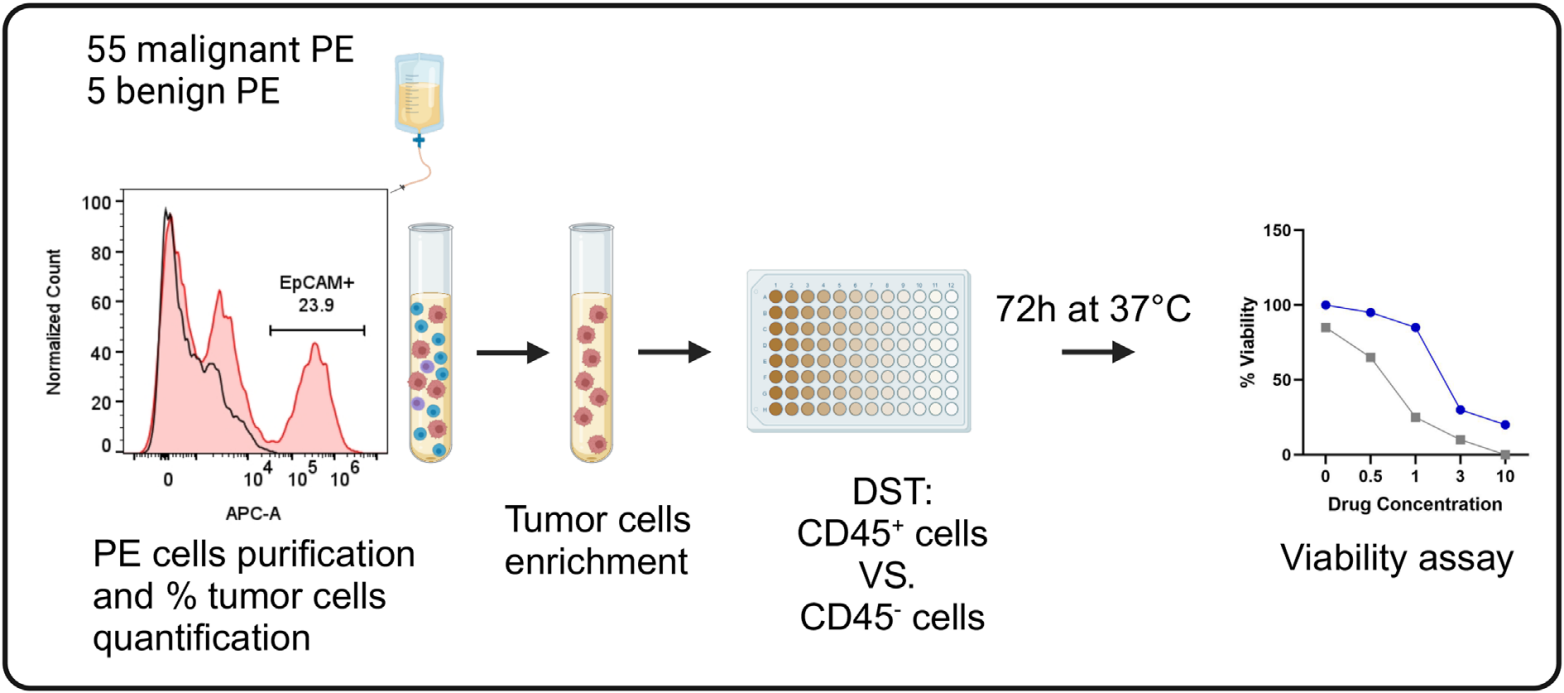
Schematic overview of the workflow for personalized medicine utilizing tumor cells from malignant pleural effusions. Malignant pleural effusions (PE) are drained, and the cells are collected and processed: cells are pelleted, red blood cells (RBCs) are lysed, and the remaining cells are screened for epithelial cell adhesion molecule (EpCAM) expression. Only effusions containing EpCAM^+^ cells continue for tumor cell enrichment. Following tumor cell enrichment, cells are then dispensed with targeted drugs at different concentrations into 96‐well plates and cultured for 72 h. Subsequently, a viability assay is performed to determine the most potent drug for treatment recommendation. The entire process, from MPE drainage to treatment recommendation, is completed within 96 h. Finally, the drug sensitivity testing (DST) result is compared with clinical outcomes. The figure was created with Biorender.com.

The overall goal of this study was to develop a robust system for personalized DST in NSCLC by utilizing MPEs (Fig. 1). The first requirement for this process is the rapid identification of tumor cells in the sample. EpCAM identification by flow cytometry was previously shown to discriminate between MPEs and effusions secondary to benign conditions [16]; it is not used in current clinical practice. Here, we validated this method, testing a total of 60 pleural effusions that were drained from 55 cancer patients and five patients experiencing benign conditions. Their age, gender, diagnosis, and cytologic results are summarized in Table 1, and a complete list is provided in Table S1.

**Table 1:** Patients' clinical characteristics.

Characteristics	Patients (*n* = 60)
Age	
Median	70
Range	38–89
Gender	
Female	45 (75%)
Male	15 (25%)
Diagnosis	
CML	1 (1.8%)
Sarcoma	1 (1.8%)
Ovarian carcinoma	8 (13.3%)
Breast adenocarcinoma	5 (8.3%)
Colorectal carcinoma	1 (1.8%)
Lung adenocarcinoma	38 (63.3%)
Lung small cell carcinoma	1 (1.8%)
Chronic renal failure	2 (3.3%)
Chronic heart failure	1 (1.8%)
Liver cirrhosis	1 (1.8%)
Meig's syndrome	1 (1.8%)
Cytology	
Negative	13 (21.6%)
Positive	47 (78.4%)

**Fig. 2:**
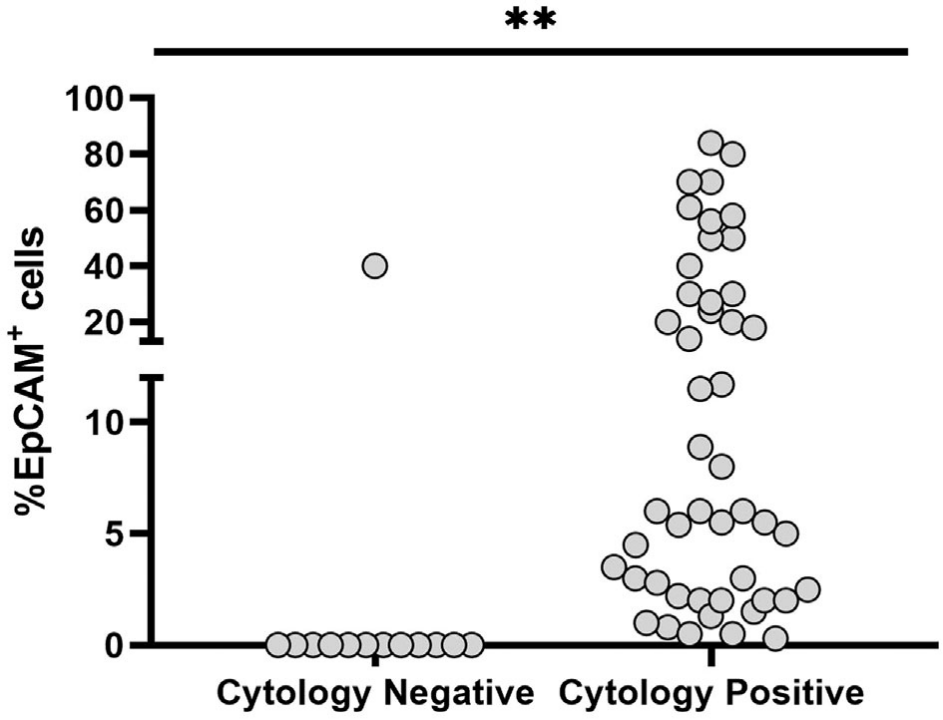
Percentage of EpCAM^+^ cells correlates with clinical cytology results. Pleural effusion %EpCAM^+^ cells determined by flow cytometry analysis in cytological examination negative and positive groups (*n* = 13 or 47). Median percentage of EpCAM^+^ cells was significantly higher in the samples with positive cytological results (**P < 0.01, Mann–Whitney test). EpCAM, epithelial cell adhesion molecule.

Indeed, as previously shown [16], EpCAM expression was detected almost exclusively in MPEs (Table 1). Moreover, it highly correlated with positive cytological examination results (Fig. 2), showing 100% sensitivity compared with cytology. Importantly, EpCAM was not identified in cells purified from pleural effusions of patients with benign conditions, nor from cancer patients whose cytological examination was negative for malignancy (Table S1).

The only exception was a single patient that had negative cytology while having 40% EpCAM^+^ cells (Fig. 2, patient 14 in Table S1). This patient suffered from metastatic NSCLC and dementia and did not receive any specific oncologic treatment. Her previous cytology result was positive, and therefore, her effusion was considered to be positive for the sake of specificity calculation. Thus, it can be concluded that EpCAM flow cytometry showed 100% specificity and sensitivity for the detection of epithelial tumor cells in MPEs, when compared with clinical cytology.

### 3.2 Direct tumor cells isolation methods result in low tumor cell yield

While EpCAM FACS‐based analysis allowed rapid tumor cell detection, it also demonstrated that tumor cells usually represent only a minority of MPE cells. Thus, it is necessary to establish a method for their enrichment before performing DST. Several methods were tested and compared in the present study, and they are summarized in Table 2.

**Table 2:** Summary of the enrichment methods tested.

	pluriStrainer®	MACS® cell separation	Mojo Sort™ cell separation
Manufacturer	pluriSelect	Miltenyi Biotec	BioLegend
Method principle	Cell diameter‐based separation	Cell‐surface antigen immunomagnetic separation	Cell‐surface antigen immunomagnetic separation
Selection	Positive by tumor cells diameter	Positive by anti‐EpCAM magnetic beads	Negative by anti CD45 magnetic beads
Time	~ 5 min	1–2 h	20–30 min
%EpCAM^+^ cells – recovery rate	Moderate	Low	High
%EpCAM^+^ cells – fold increase	Low	Low	High

**Fig. 3:**
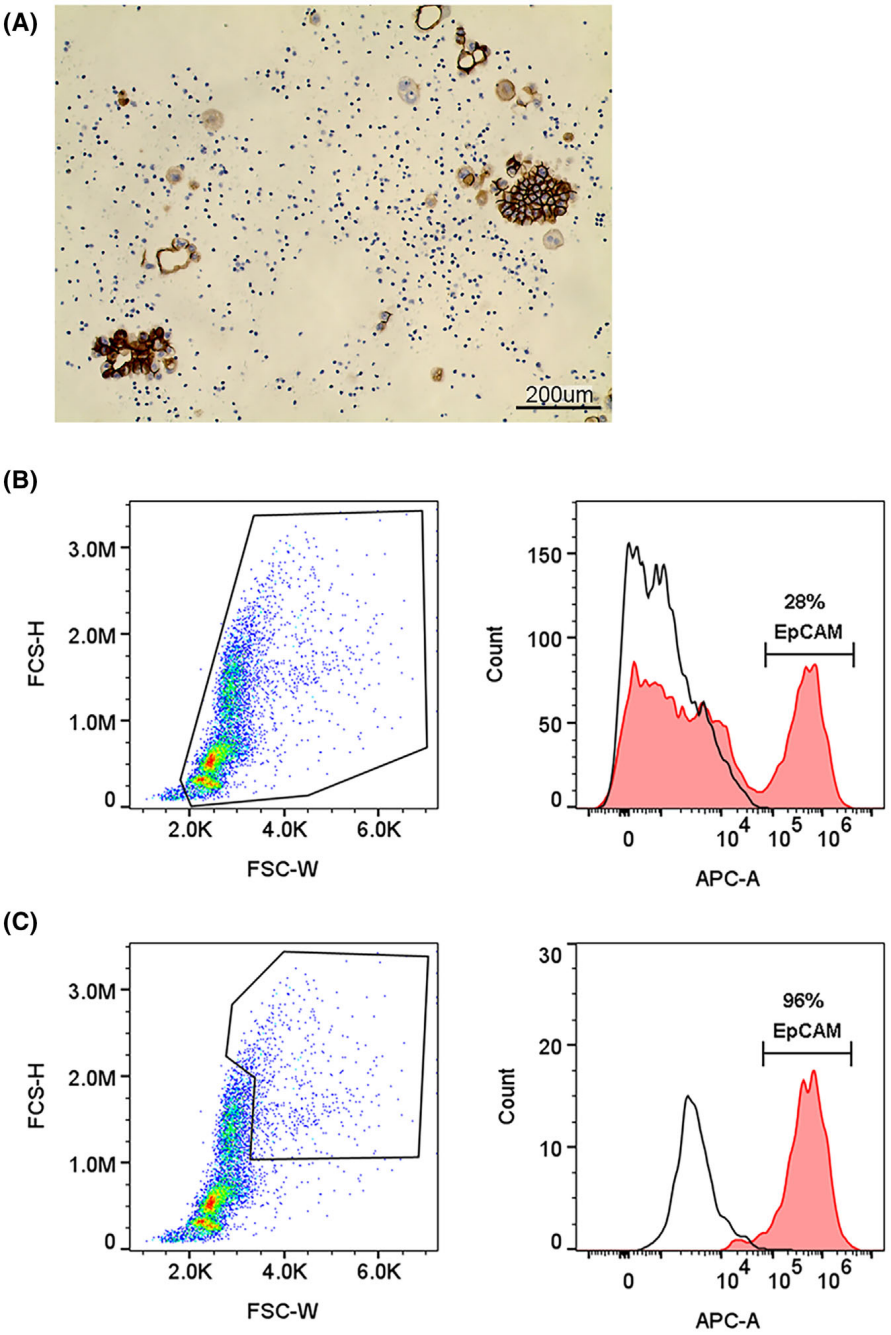
Most of the cell aggregates from malignant pleural effusions (MPEs) are tumor cells. (A) Representative immunohistochemistry of cells purified from MPE stained with anti‐EpCAM antibody. Scale bar, 200 μm. (B) Flow cytometry analysis of cells isolated from MPE shows the gating strategy and histograms of EpCAM staining. Representative upper panels show the percentage of tumor cells (EpCAM^+^) in the total cells' population, while (C) lower panels show the percentage of tumor cells in the top 5% of the cells' population according to size. All images and panels are representative of three different samples of MPEs.

One parameter which is highly notable in tumor cells within MPEs is that their cell diameter is larger than that of other cell types identified in the fluids, and they often form clusters [22] (Fig. 3A). Indeed, when gating the top 5% largest cells from a sample of MPE that is composed of 28% EpCAM^+^ cells (Fig. 3B), the majority of the gated cells are EpCAM^+^ (Fig. 3C).

Furthermore, it was previously shown that ovarian tumor cells from malignant ascites can be purified by passing the cells through a nylon mesh filter and collecting the large cell clumps that remained trapped [23].

**Fig. 4:**
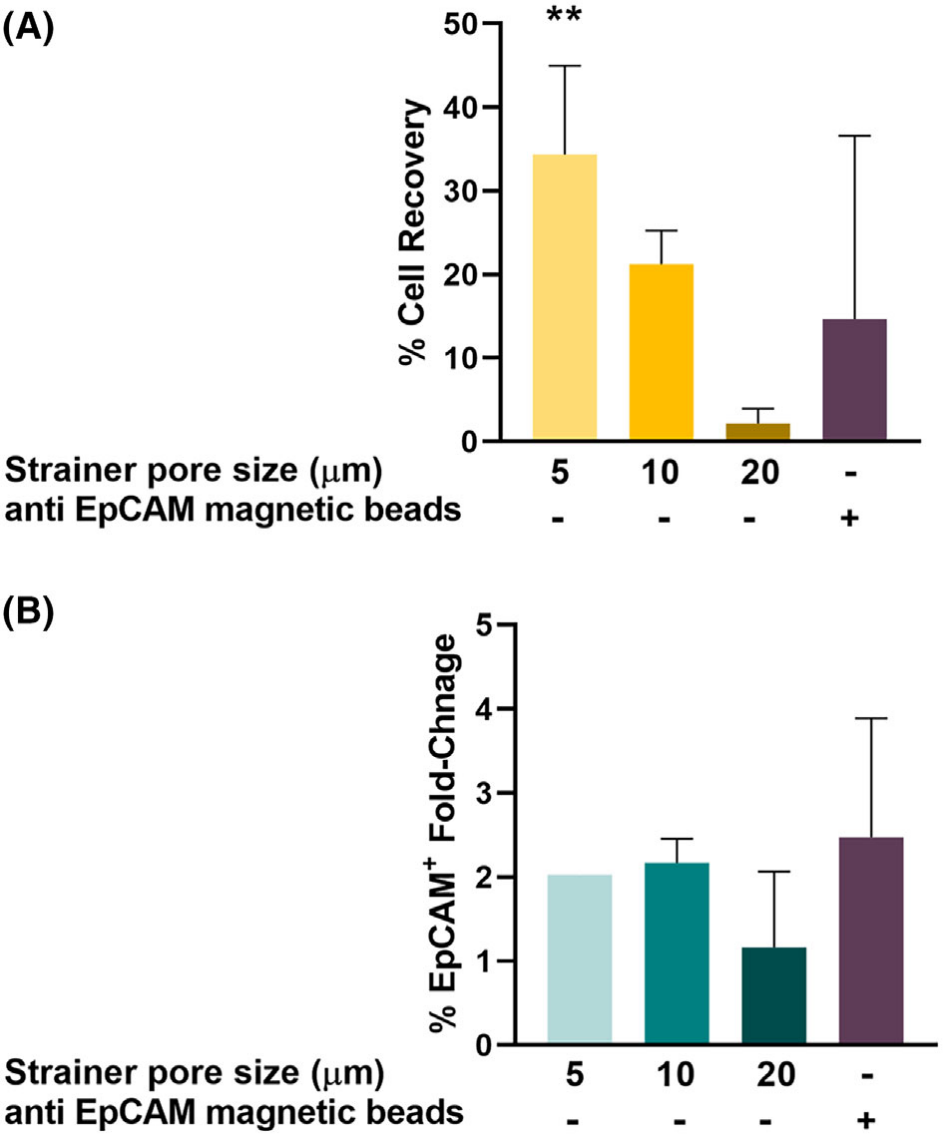
Tumor cells enrichment by anti‐epithelial cell adhesion molecule (EpCAM) magnetic beads and filtration of malignant pleural effusion (MPE) through a cell strainer. Comparison of tumor cells (EpCAM^+^) isolation efficiency (% cell recovery) (A) and tumor cells enrichment (%EpCAM^+^ fold change) (B), using anti‐EpCAM magnetic beads and 5‐, 10‐ or 20‐μm pore‐size strainers. Percentage of cell recovery is calculated by dividing the number of live EpCAM^+^ cells following enrichment by the initial number of live EpCAM^+^ cells, multiplied by 100. Tumor cell enrichment is calculated by dividing % EpCAM^+^ cells following enrichment by the initial % EpCAM^+^ cells. *n* = 3. **P ≤ 0.01. Statistical significance was evaluated using a Welch's ANOVA test. Error bars indicate standard errors.

Hence, to employ a similar method, a range of cell strainers with different pore sizes (5, 10, and 20 μm) were used to capture tumor cells from MPEs. When a strainer with a 20‐μm mesh was utilized, the cell recovery rate was lower compared with the 10 and especially the 5‐μm mesh (Fig. 4A). Evidently, the 5‐μm mesh managed to capture tumor cells in the most efficient manner, yielding an average cell recovery rate of 34% (Fig. 4A). However, a twofold tumor cell enrichment was observed with all pore sizes (Fig. 4B).

Thus, although diameter‐based separation of MPE tumor cells is a straightforward and rapid approach, it is not adequate as it fails to yield a sufficient concentration of tumor cells for subsequent DST, especially for MPEs with low tumor cell percentage, as only a twofold enrichment was observed.

Since EpCAM is a sensitive and specific tumor marker in the context of MPEs, we next employed immunomagnetic‐based cell isolation as an alternative enrichment method, utilizing anti‐EpCAM antibody‐coated magnetic beads to capture and isolate tumor cells from MPEs.

Similar to size‐based separation, EpCAM^+^ cell isolation enriched tumor cells by only an average of 2.5‐fold (Fig. 4B), and the recovery rate was low as well, averaging 14.5% (Fig. 4A), implying that a significant number of the initial EpCAM^+^ cells were lost during the procedure. Hence, utilizing EpCAM magnetic beads for positive selection also proved inefficient for tumor cell enrichment from MPEs.

### 3.3 CD45 ^+^ cell depletion efficiently enriches tumor cells from MPEs

Since both direct isolation methods proved ineffective, with low tumor cell recovery and enrichment, we hypothesized that indirect isolation through nontumor cell depletion may result in higher yields of tumor cells.

**Fig. 5:**
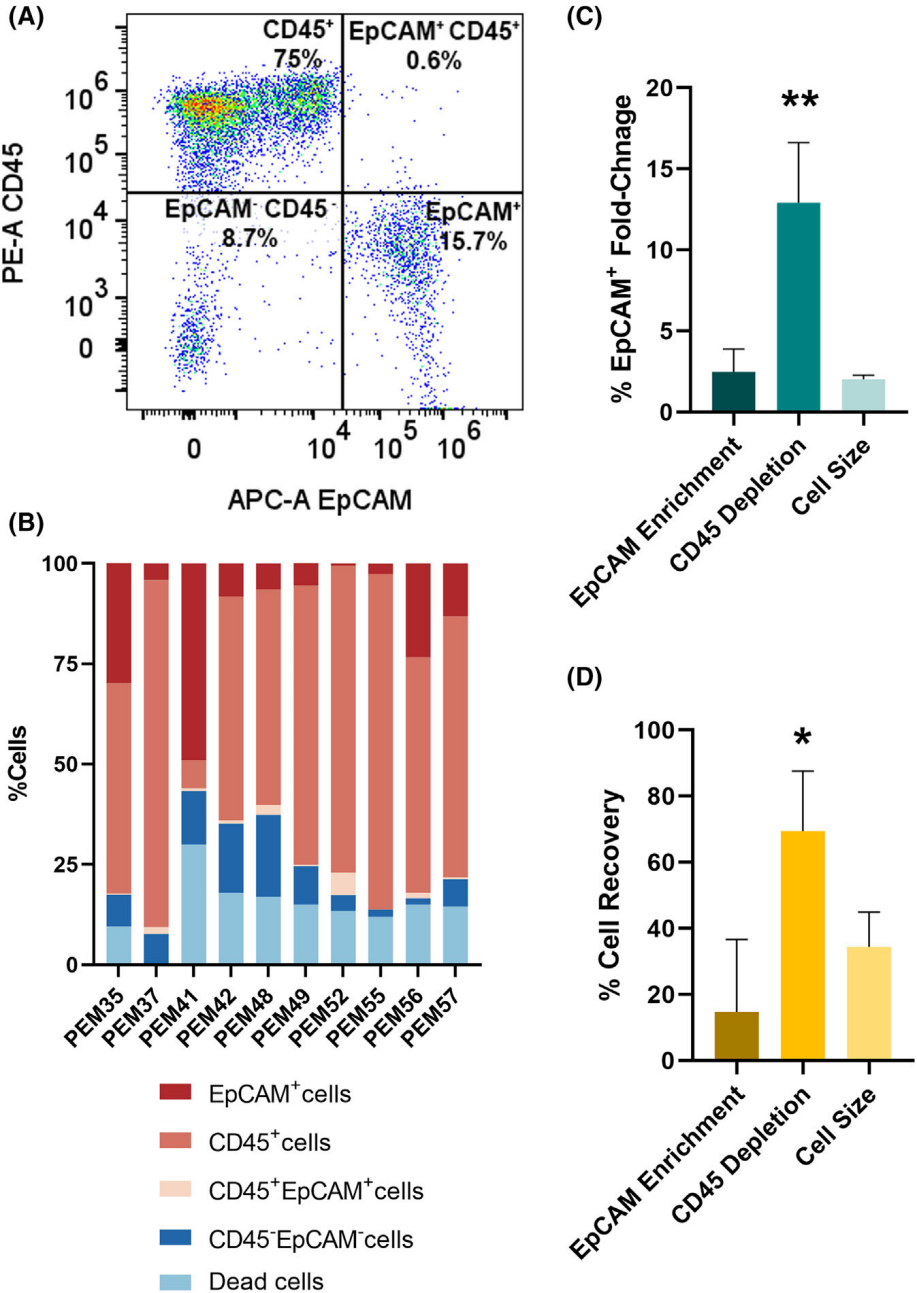
Depletion of abundant CD45^+^ cells is an efficient method for indirect enrichment of tumor cells from malignant pleural effusions (MPEs). (A) Representative flow cytometry panel showing CD45 and epithelial cell adhesion molecule (EpCAM) staining of MPE cells, following red blood cell (RBC) lysis. (B) Stacked bar graph showing the distribution of tumor cells and immune cells within MPE samples collected from cancer patients. Comparison of tumor cell enrichment (C) and (D) tumor cell (EpCAM^+^) isolation efficiency (% cell recovery) between two immunomagnetic bead‐based methods (EpCAM‐positive enrichment, CD45 depletion) and cell size separation using a 5‐μm strainer. Percentage of cell recovery is calculated by dividing the number of live EpCAM^+^ cells following enrichment by the initial number of live EpCAM^+^ cells, multiplied by 100. Tumor cell enrichment (%EpCAM^+^ fold change) is calculated by dividing % EpCAM^+^ cells following enrichment by the initial % EpCAM^+^ cells. *n* = 3, **P ≤ 0.01, *P ≤ 0.05. Statistical significance was evaluated using a one‐way ANOVA followed by Tukey's test. Error bars indicate standard errors.

As alluded to above, tumor cells usually represent a fraction of the total MPE cell population, which includes various cell types like leukocytes and mesothelial cells [18, 24, 25]. Thus, we utilized flow cytometric analysis, using antibodies targeting EpCAM (tumor cells [16]) and CD45 (pan‐leukocyte marker [26]) to distinguish between tumor cells and immune cells within MPE samples. The analysis revealed that these two cell types constituted the predominant populations within MPEs, with minimal detectable overlap between them (Fig. 5A). Interestingly, a distinct population of cells lacked expression of both EpCAM and CD45 (CD45^−^/EpCAM^−^), reflecting the presence of additional cell types, such as mesothelial cells, within MPEs.

Furthermore, analysis of nine MPE samples collected from lung cancer patients revealed a consistent pattern of cellular distribution. Immune cells represented the predominant cell population, while tumor cells comprised a smaller fraction of the total cell count (Fig. 5B), accounting together for ~ 90% of the total live cell population. The CD45^−^/EpCAM^−^ cell population consistently represented up to 10% of the total cell population. Notably, in one sample (PEM52), collected from an ovarian cancer patient, the majority of EpCAM^+^ cells were also positive for CD45.

Given the abundance of CD45^+^ cells in lung cancer MPE samples, the use of anti‐CD45 magnetic beads was explored to deplete leukocytes from MPEs, thereby enriching the remaining tumor cell population.

Depletion of CD45^+^ cells led to a significant enrichment of EpCAM^+^ cells (Fig. 5C). Notably, more than 50% of the tumor cells were recovered following depletion, a rate that is significantly higher than that obtained by EpCAM‐positive selection and size‐based enrichment strategies (Fig. 5D).

Overall, separation of tumor cells using CD45^+^ cell depletion proved to be the most efficient and effective method among those tested in this study.

### 3.4 Enhanced tumor cell enrichment by repeated CD45 ^+^ cell depletions

**Fig. 6:**
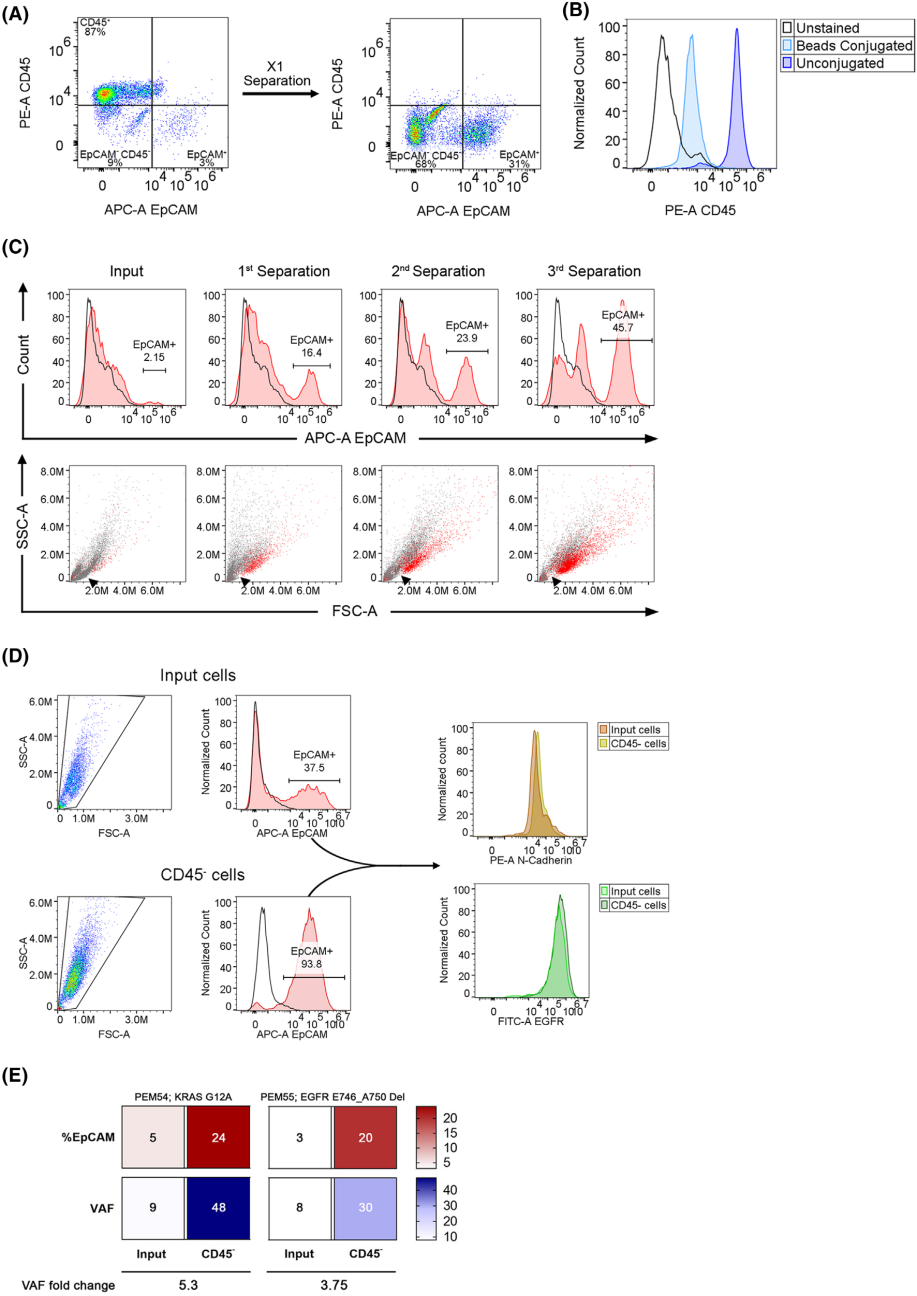
CD45 repeated magnetic separations significantly improve tumor cell enrichment from malignant pleural effusions (MPEs). (A) Representative flow cytometry panels demonstrating tumor cell enrichment (EpCAM^+^/CD45^−^) following one round of CD45 depletion. (B) Representative CD45 flow cytometry histograms showing a significant reduction of the MFI (median fluorescence intensity) of MPE cells following incubation with anti‐CD45 magnetic beads (‘Beads conjugated’ versus ‘Unconjugated’). (C) Representative EpCAM flow cytometry histograms (upper panels) from repeated magnetic separations utilizing anti‐CD45 antibody‐coated magnetic beads and forward scatter‐side scatter (FSC‐SSC) dot plots (lower panels) of the same cell population. Red dots represent EpCAM^+^ cells, and arrowheads point to an area of depleted cell population. (D) Representative gating strategy and EpCAM flow cytometry histograms of input and CD45^−^ cells (two rounds of magnetic separations utilizing anti‐CD45 antibody‐coated magnetic beads), followed by EGFR and N‐Cadherin histograms obtained from EpCAM^+^ gated populations. (E) Targeted next‐generation sequencing of KRAS mutation or EGFR deletion was performed on MPE input and CD45^−^ cells (two rounds of magnetic separations utilizing anti‐CD45 antibody‐coated magnetic beads) from two patients. Heat maps representing percentages of tumor cells and variant allele frequencies (VAF). All panels are representative of three different samples of MPEs. EpCAM, epithelial cell adhesion molecule.

CD45 depletion using anti‐CD45 magnetic beads resulted in a significant increase in tumor cell purity within MPE samples, elevating the tumor cell percentage by ~ 10‐fold. However, a considerable nontumor and nonleukocyte cell (CD45^−^/EpCAM^−^) population was identified following depletion (Fig. 6A), indicating the need for further optimization to achieve optimal tumor cell isolation.

The identity of the CD45^−^/EpCAM^−^ cells' population that was observed following CD45 depletion could be any of the following cells: mesothelial cells, platelets, and potentially CD45^+^ cells that were not detected by anti‐CD45 antibodies, possibly due to low expression of C45. Interestingly, the addition of anti‐CD45 coated magnetic beads to an MPE sample, without subsequent magnetic separation, led to a reduction in the apparent CD45^+^ cell population according to FACS analysis (Fig. 6B). This finding suggests that the magnetic beads may sterically block the binding of anti‐CD45 antibodies to their target epitopes, resulting in an underestimation of CD45^+^ cell numbers. Thus, the CD45^−^/EpCAM^−^ cell population may be comprised of leukocytes that are bound to magnetic beads. Therefore, additional rounds of magnetic separations may be necessary in order to enhance the efficiency of CD45 depletion and tumor cell enrichment. Indeed, a significant increase in EpCAM^+^ tumor cell percentage was observed after three rounds of depletion (Fig. 6C). Furthermore, the increase in the number of relatively large EpCAM^+^ cells (red‐labeled cells in the lower panels of Fig. 6C) was correlated to a reduction in the number of relatively smaller cell population (arrowheads, Fig. 6C), suggesting a successful removal of immune cell subsets by repeated magnetic separations.

Notably, unlike CD45 beads, the addition of anti‐EpCAM coated magnetic beads to an MPE sample, without subsequent magnetic separation, did not lead to a reduction in the apparent EpCAM^+^ cell population according to FACS analysis (Fig. S1), implying that the steric block by magnetic beads is dependent on the specific antibody clones that are used for the analysis.

Additionally, to verify that tumor heterogeneity isn't altered by the enrichment, we assessed the expression of two cellular markers, EGFR and N‐Cadherin [27, 28], in the original MPE sample and compared it to the tumor cell‐enriched sample following two rounds of CD45^+^ cells depletion. Indeed, while there was a significant increase in EpCAM^+^ cells percentage following depletion, the expression pattern of the markers was unaltered (Fig. 6D). Furthermore, targeted next‐generation sequencing (NGS) analysis of two MPE samples, one harboring the KRAS G12A mutation and a second harboring the EGFR exon 19 Glu746‐Ala750 deletion, revealed an increase in the mutated allele frequency following CD45^+^ cells depletion, correlating with EpCAM^+^ tumor cell enrichment (Fig. 6E). These findings validate that repeated CD45^+^ depletions enrich tumor cells that are representative of the original tumor cell population and can increase tumor cell percentage to ~ 50% or more, thereby allowing drug sensitivity evaluation to be performed on a majority of tumor cells.

### 3.5 DST of tumor cells enriched from MPE is predictive of clinical response

**Fig. 7:**
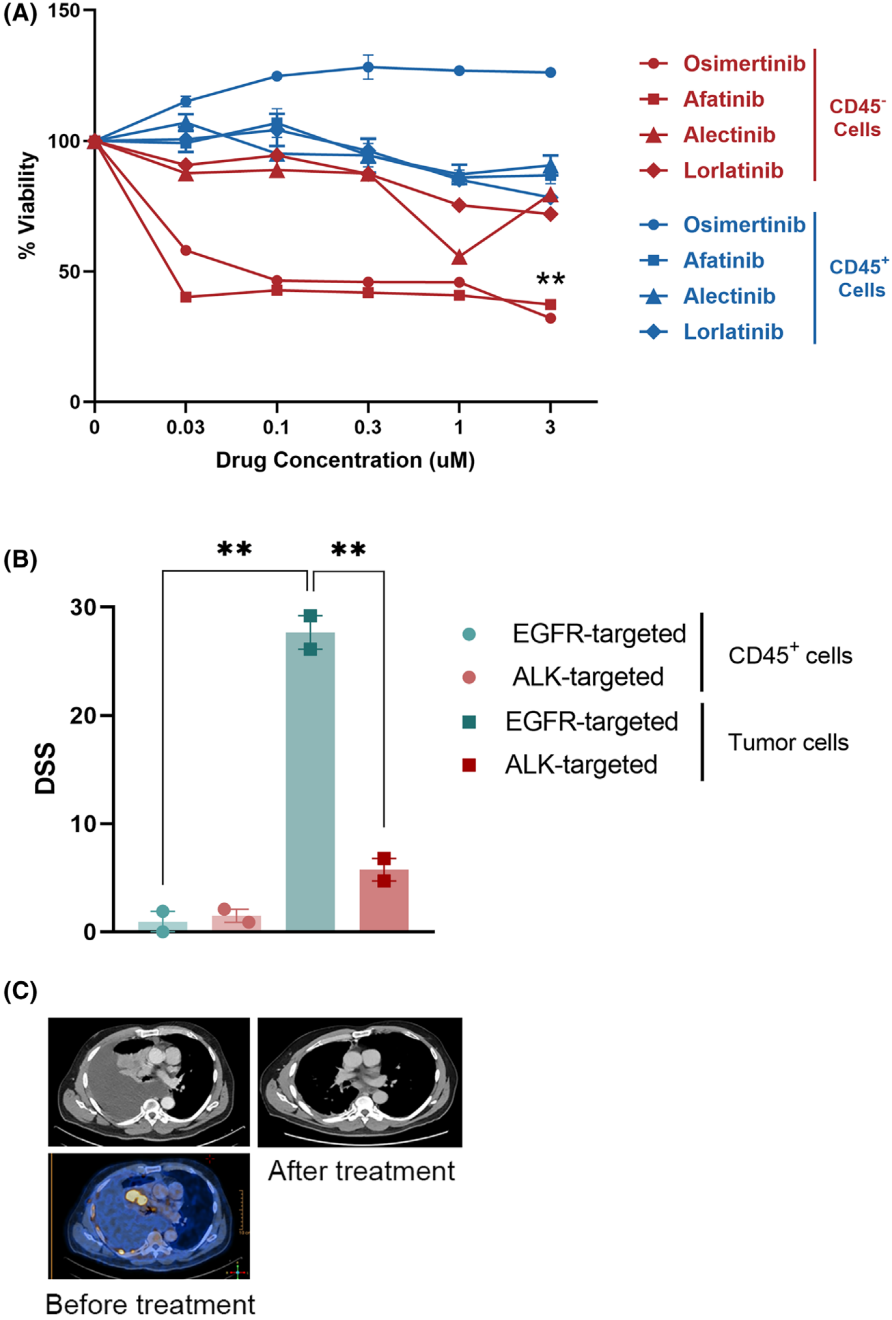
Drug sensitivity testing of malignant pleural effusion (MPE) CD45^−^ cells predicts genotype‐matched therapeutic response. (A) Dose–response curves of osimertinib, afatinib, alectinib, and lorlatinib in CD45^+^ and CD45^−^ cells, isolated from epidermal growth factor receptor (EGFR)‐driven non‐small cell lung cancer MPE. *n* = 4, **P ≤ 0.01 for osimertinib and afatinib when comparing CD45^−^ to CD45^+^ cells. Statistical significance was evaluated using an unpaired *t*‐test. Error bars indicate standard errors. (B) Drug sensitivity score (DSS) calculated for (A), comparing between CD45^+^ and CD45^−^ cells and between EGFR (osimertinib and afatinib) and anaplastic lymphoma kinase (ALK) targeted drugs (alectinib and lorlatinib), **P ≤ 0.01. Statistical significance was evaluated using an unpaired *t*‐test. Error bars indicate standard errors. (C) Computed tomography (CT) and positron emission tomography (PET‐CT) scans of the same patient before and after 3 months of osimertinib treatment. The pleural effusion and two nodules in the right lung disappeared.

Following the establishment of an efficient tumor cell enrichment procedure, we aimed to assess the predictive validity of DST results when employing CD45^+^ depletion prior to DST. This was achieved by testing the response of tumor cells, which were isolated from an MPE sample of a treatment‐naïve EGFR‐driven NSCLC patient, to targeted treatments and followed by correlation with genetic alterations and clinical outcomes. The sample was subjected to CD45^+^ cell depletion and the isolated tumor cells (CD45^−^ cells) were treated with a panel of targeted drugs at varying concentrations for 72 h, followed by cell viability assessment using MTS assay to determine drug efficacy. To assess the impact of the drugs on control immune cells, the CD45^+^ cell population (immune cells) underwent the same test and was compared with the CD45^−^ cell population (tumor cells). Indeed, DST revealed a significantly enhanced sensitivity to targeted therapies within the CD45^−^ cell population compared with the CD45^+^ population (DSS > 10, Fig. 7A,B). These findings emphasize the importance of isolating tumor cells by depletion of CD45^+^ from heterogeneous MPE samples for accurate drug efficacy evaluation. Additionally, differential drug responses were observed within the CD45^−^ cell population, with greater sensitivity to EGFR inhibitors (osimertinib, afatinib) compared with ALK inhibitors (alectinib, lorlatinib) (DSS > 10, Fig. 7A,B). Indeed, in alignment with DST results, a clinical partial response was observed following 3 months of treatment with osimertinib (Fig. 7C). These findings emphasize the potential use of enriched tumor cells from MPE samples for DST to guide personalized treatment decisions in NSCLC.

## 4 Discussion

Lung cancer remains a leading cause of cancer‐related mortality, with NSCLC representing the largest subset. While targeted therapies have improved outcomes for patients with specific driver mutations, the emergence of resistance mechanisms necessitates a more comprehensive approach to treatment selection. Furthermore, the evolving mutational patterns of NSCLC and the limitations of current genetic testing emphasize the need for unbiased drug screening platforms to identify optimal therapeutic strategies for individuals.

Drug sensitivity testing is a promising tool for personalized medicine in NSCLC, but its implementation in treatment guidance is hindered by several critical factors. Prolonged turnaround times associated with methods that require the establishment of tumor organoids or pure cell lines [5, 12] and the potential for phenotypic and genetic alterations of tumor cells during extended culture limit its clinical utility [29, 30]. Additionally, overgrowth of nontumor cells in the tumor microenvironment can obscure the response of tumor cells to drugs [10, 31].

To address these limitations, we focused on developing a rapid and efficient method for isolating tumor cells from MPEs for further DST, as MPEs can serve as a rich source for tumor cells.

First and foremost, a reliable method for identifying tumor cells within MPEs is essential. We utilized EpCAM expression as a biomarker to identify tumor cells within MPEs using flow cytometry. Our results demonstrated the high sensitivity and specificity of EpCAM for detecting tumor cells, supporting its integration as a valuable diagnostic tool.

Given the low median percentage of tumor cells in MPEs, we evaluated strategies for enriching tumor cells in order to ensure that subsequent DST results primarily reflected tumor cell response.

While some research groups have utilized EpCAM‐based tumor cell enrichment from malignant effusions for DST [10], and others have used CD45^+^ cell depletion to enrich for tumor cells before DNA sequencing [32], no study has compared different tumor cell isolation methods from malignant effusions to reach optimal tumor cell recovery and tumor cell enrichment. This is particularly critical for DST in which a high number of live tumor cells is required to simultaneously test their sensitivity to hundreds of drugs and drug combinations in parallel on the same sample.

The first method that was tested exploited the distinct size difference between nontumor cells and tumor cells in MPE, which tend to be larger and often form clusters [22, 33, 34]. Utilizing filters with different pore sizes to selectively retain the larger tumor cells, the 5‐μm filter demonstrated the highest cell recovery rate. However, the overall enrichment of EpCAM^+^ tumor cells remained suboptimal. This was possibly because the 5‐μm filter retained both tumor and nontumor cells, resulting in a low tumor cell enrichment factor similar to filters with larger pore sizes. These findings suggest that while size‐based filtration can contribute to tumor cell enrichment, additional strategies are necessary to achieve the desired purity levels, particularly in MPEs with low tumor cell burdens.

Next, tumor cell enrichment was assessed by techniques in which specific cell populations are isolated using antibody‐coated magnetic beads. Given the sensitivity and specificity of EpCAM‐based tumor cell identification, an initial strategy focused on EpCAM‐based positive selection. However, the low recovery rates and suboptimal enrichment achieved using this method necessitated alternative approaches.

MPEs are characterized by a heterogeneous cellular composition, primarily composed of immune cells (CD45^+^) and tumor cells (EpCAM^+^). Importantly, we have found minimal overlap between CD45 and EpCAM staining in lung cancer samples. Thus, to circumvent the limitations of EpCAM‐based enrichment, a CD45 depletion strategy was evaluated. By targeting the ubiquitous expression of CD45 on leukocytes, this approach enabled a significant enrichment of tumor cells, demonstrating superior efficacy compared with EpCAM‐based selection alone. Importantly, multiple rounds of CD45 depletion further increased tumor cell purity and reduced nontumor cell populations. While CD45^+^ cells represent the major cell population in MPEs, roughly 10–20% of MPE cells were CD45^−^/EpCAM^−^ cells, possibly representing mesothelial cells. Depleting mesothelial cells presents a challenge due to the lack of specific surface markers. However, the relatively low abundance of CD45^−^ nontumor cell populations suggests that a CD45 depletion strategy alone may be sufficient to achieve a tumor cell‐enriched population in most of the samples.

Interestingly, several groups have compared tumor cell isolation techniques for circulating tumor cells (CTC) from the blood. Saini et al showed that Parsortix® PR1 (size‐ and deformability‐based enrichment) was optimal for CTC enrichment [35], while Drucker et al. showed that the ScreenCell® (size‐based enrichment) method was optimal [36]. Comparing with data presented here, these studies pointed to size‐based tumor cell isolation methods as optimal, while we found CD45^+^ cell depletion as optimal. This difference may be related to the different cell concentrations in malignant effusions compared with blood samples, as tumor cells are rare in blood (1–1000 in a standard 5‐mL blood tube) [35], while CD45^+^ cells are abundant. Thus, CD45 depletion may not be practical for blood samples in which the tumor/CD45^+^ cell ratio is ~ 1 : 10^6^. On the contrary, the large number of tumor cells, including tumor cell aggregates, in MPEs may clog the pores of filters used for tumor cell isolation, reducing the efficiency of size‐based methods for MPEs. Similarly, the low tumor cell isolation efficiency that was found here for immunomagnetic enrichment of tumor cells using EpCAM (MACS cell separation system, Miltenyi Biotec) may be the result of tumor cell aggregates clogging the MACS columns used with this technology.

Finally, to evaluate the predictive accuracy of DST in guiding therapeutic decisions for lung cancer patients, tumor cells were isolated from a treatment‐naive EGFR‐mutant NSCLC patient using the optimized CD45^+^ cell depletion enrichment method. The isolated tumor cells were subjected to a panel of targeted therapies, demonstrating differential sensitivity profiles. Notably, the CD45^−^ tumor cell population exhibited enhanced sensitivity to EGFR inhibitors compared with the CD45^+^ immune cell population, highlighting the importance of isolating pure tumor cell populations for accurate drug response assessment. Importantly, the observed *in vitro* sensitivity to osimertinib correlated with clinical response.

The successful application of DST to a treatment‐naive EGFR‐mutant NSCLC patient emphasizes the potential of utilizing DST combined with advanced cell isolation techniques in guiding personalized treatment strategies for NSCLC patients.

While this study provides a foundation for developing robust DST platforms, further research is needed to explore its applicability across diverse cases where targeting EpCAM for identification of tumor cells may not be suitable.

First, although EpCAM was found to be a reliable marker for tumor cells in blood, malignant ascites, and MPE, there are cases in which it fails to identify tumor cells. Heterogeneity of the tumor or changes in cellular phenotype, as epithelial‐to‐mesenchymal transition (EMT), can diminish EpCAM expression or impede its detection [22, 37]. Furthermore, EpCAM expression is limited in rare malignancies with distinct cellular origins, including sarcomas and mesotheliomas [38]. Consequently, while EpCAM fulfills the specificity and rapidity requirements of our methodology, alternative diagnostic strategies are necessary for these specific cases. Indeed, several studies have explored the utility of markers, such as CEA, CDH1, and MGB1 for CTC identification in cancer patient blood [27, 39]. Moreover, de Wit et al. were able to identify that there are CTCs within the EpCAM^−^ population of cells using Pan‐Cytokeratin antibodies [40]. Similarly, in MPEs, markers like CYFRA 21‐1, CEA, and NSE have proven to be effective for tumor cell detection [41, 42].

Proteomic approaches were also utilized to characterize and discover alternative surface markers for tumor cells in MPEs [43, 44].

Finally, targeted next‐generation sequencing (NGS) can contribute to the characterization of tumor cell driver mutations in MPEs [45] and, as our study has demonstrated, can corroborate tumor cell identification following enrichment.

A second limitation of this study arises in cases in which performing repeated CD45^+^ cell depletion does not contribute to tumor cell enrichment and integrity. We identified one case of MPE, originating from an ovarian cancer patient, where a substantial proportion of the EpCAM^+^ cells were co‐stained for CD45. There are some indications in the literature of EpCAM^+^/CD45^+^ cell populations. It has been demonstrated that in ascites fluid samples of ovarian cancer patients, especially in postchemotherapy samples, the majority of tumor cells are CD45^+^/EpCAM^+^ cells derived from the EpCAM^+^ primary tumor site. These cells displayed high resistance to therapy and increased invasiveness compared with the CD45^−^/EpCAM^+^ cell population [38].

The presence of CD45^+^/EpCAM^+^ cells was also reported in solid tumor tissues, MPEs, and blood of patients with NSCLC [46]. These doubly positive cells were highly suspected to undergo EMT, potentially enabling them to better infiltrate into the PE and blood [47].

Moreover, EpCAM^+^ CTCs from colorectal cancer have been reported to fuse with macrophages to form CD45^+^ circulating hybrid cells, which exhibited increased tumor heterogeneity and metastatic behavior [48].

Thus, in cases where EpCAM^+^ cells co‐express CD45, the efficacy of repeated CD45^+^ cell depletion may be compromised. Furthermore, the exclusion of the EpCAM^+^/CD45^+^ cell subpopulation from the depleted cell fraction will not preserve the original MPE tumor heterogeneity and will fail to reliably reflect its drug sensitivity. To address this limitation, alternative methods for cell depletion, such as the use of different immune cell markers, for instance—a combination of CD3 and CD19 for lymphocytes and CD11b for macrophages depletion, which have been found to be abundant in MPE [49], should be explored.

## 5 Conclusions

Considering treatment resistance that rises following 1–2 years of targeted therapy in NSCLC, DST is required to help in the treatment decision on the best next‐line treatment. To test the sensitivity of tumor cells, one must first isolate them. In this study, several tumor cell isolation techniques were compared for their efficiency in enriching tumor cells from MPEs: immunomagnetic enrichment of epithelial cells using EpCAM (MACS cell separation system, Miltenyi Biotec), negative selection via immunomagnetic CD45^+^ cell depletion (MojoSort™ Human CD45 Nanobeads, BioLegend), and size‐based separation of tumor cells utilizing cell strainers (pluriStrainer®). Negative selection via immunomagnetic CD45^+^ cell depletion was the most efficient tumor cell isolation technique, showing the highest tumor cell recovery rate and the highest tumor cell fold enrichment. Furthermore, by utilizing repeated rounds of magnetic separation, this method produced samples in which tumor cells represented the majority of the cells, even in samples that had an initial low tumor cell percentage (2–5%). Importantly, the results of the DST performed on the enriched samples correlated with clinical outcomes. Thus, the method presented here holds significant promise for facilitating rapid and reliable treatment decisions for NSCLC patients, paving the way to personalized therapeutic approaches.

## Conflict of interest

NM, SC, LB, AO, and MP filed a patent related to the development of the method described.

## Author contributions

MP and NM contributed to the conception, design of the work, acquisition, analysis, and interpretation of the data. HB, IK, OZ, NH, and OW contributed to the acquisition and analysis of the data. SC and AO contributed to the acquisition of the data. JB and LB contributed to the interpretation of the data. All authors reviewed the manuscript, edited the manuscript, and provided substantial feedback for the study.

## Supporting information


**Fig. S1.** Anti‐EpCAM magnetic beads don't block the binding of anti‐EpCAM antibodies to their target epitopes.

**Table S1.** Patients' clinical characteristics and %EpCAM^+^ in their MPE cells.

## Data Availability

The data that support the findings of this study are available in the figures, tables, and the supplementary material of this article.

## References

[1] Pao W , Girard N . New driver mutations in non‐small‐cell lung cancer. Lancet Oncol. 2011;12(2):175–180. 21277552 10.1016/S1470-2045(10)70087-5

[2] Chevallier M , Borgeaud M , Addeo A , Friedlaender A . Oncogenic driver mutations in non‐small cell lung cancer: past, present and future. World J Clin Oncol. 2021;12(4):217–237. 33959476 10.5306/wjco.v12.i4.217 PMC8085514

[3] Tan AC , Tan DSW . Targeted therapies for lung cancer patients with oncogenic driver molecular alterations. J Clin Oncol. 2022;40(6):611–625. 34985916 10.1200/JCO.21.01626

[4] Sato H , Offin M , Kubota D , Yu HA , Wilhelm C , Toyooka S , et al. Allele‐specific role of ERBB2 in the oncogenic function of EGFR L861Q in EGFR‐mutant lung cancers. J Thorac Oncol. 2021;16(1):113–126. 33038514 10.1016/j.jtho.2020.09.019 PMC7775889

[5] Kodack DP , Farago AF , Dastur A , Held MA , Dardaei L , Friboulet L , et al. Primary patient‐derived cancer cells and their potential for personalized cancer patient care. Cell Rep. 2017;21(11):3298–3309. 29241554 10.1016/j.celrep.2017.11.051 PMC5745232

[6] Herbst RS , Morgensztern D , Boshoff C . The biology and management of non‐small cell lung cancer. Nature. 2018;553(7689):446–454. 29364287 10.1038/nature25183

[7] Neel DS , Bivona TG . Resistance is futile: overcoming resistance to targeted therapies in lung adenocarcinoma. NPJ Precis Oncol. 2017;1:3. 29152593 10.1038/s41698-017-0007-0 PMC5687582

[8] Messner DA , Al Naber J , Koay P , Cook‐Deegan R , Majumder M , Javitt G , et al. Barriers to clinical adoption of next generation sequencing: perspectives of a policy Delphi panel. Appl Transl Genom. 2016;10:19–24. 27668172 10.1016/j.atg.2016.05.004 PMC5025465

[9] Planchard D , Popat S , Kerr K , Novello S , Smit EF , Faivre‐Finn C , et al. Metastatic non‐small cell lung cancer: ESMO clinical practice guidelines for diagnosis, treatment and follow‐up. Ann Oncol. 2018;29(Suppl 4):iv192–iv237. 30285222 10.1093/annonc/mdy275

[10] Ruiz C , Kustermann S , Pietilae E , Vlajnic T , Baschiera B , Arabi L , et al. Culture and drug profiling of patient derived malignant pleural effusions for personalized cancer medicine. PLoS One. 2016;11(8):e0160807. 27548442 10.1371/journal.pone.0160807 PMC4993361

[11] Ettinger DS , Wood DE , Aisner DL , Akerley W , Bauman JR , Bharat A , et al. NCCN guidelines insights: non‐small cell lung cancer, version 2.2021. J Natl Compr Canc Netw. 2021;19(3):254–266. 33668021 10.6004/jnccn.2021.0013

[12] Narasimhan V , Wright JA , Churchill M , Wang T , Rosati R , Lannagan TRM , et al. Medium‐throughput drug screening of patient‐derived organoids from colorectal peritoneal metastases to direct personalized therapy. Clin Cancer Res. 2020;26(14):3662–3670. 32376656 10.1158/1078-0432.CCR-20-0073 PMC8366292

[13] Chrabańska M , Środa M , Kiczmer P , Drozdzowska B . Lung cancer cytology: can any of the cytological methods replace histopathology? J Cytol. 2020;37(3):117–121. 33088028 10.4103/JOC.JOC_168_19 PMC7542047

[14] Clive AO , Kahan BC , Hooper CE , Bhatnagar R , Morley AJ , Zahan‐Evans N , et al. Predicting survival in malignant pleural effusion: development and validation of the LENT prognostic score. Thorax. 2014;69(12):1098–1104. 25100651 10.1136/thoraxjnl-2014-205285 PMC4251306

[15] Froudarakis ME . Pleural effusion in lung cancer: more questions than answers. Respiration. 2012;83(5):367–376. 22584211 10.1159/000338169

[16] Roca E , Lacroix R , Judicone C , Laroumagne S , Robert S , Cointe S , et al. Detection of EpCAM‐positive microparticles in pleural fluid: a new approach to mini‐invasively identify patients with malignant pleural effusions. Oncotarget. 2016;7(3):3357–3366. 26689993 10.18632/oncotarget.6581 PMC4823111

[17] Porcel JM , Esquerda A , Bielsa S , Novell A , Sorolla MA , Gatius S , et al. Epithelial cell adhesion molecule (EpCAM) from pleural fluid cell lysates is a highly accurate diagnostic biomarker of adenocarcinomatous effusions. Respirology. 2019;24(8):799–804. 30903651 10.1111/resp.13539

[18] Dong Y , Wang Z , Shi Q . Liquid biopsy based single‐cell transcriptome profiling characterizes heterogeneity of disseminated tumor cells from lung adenocarcinoma. Proteomics. 2020;20(13):1900224. 10.1002/pmic.201900224 31960581

[19] Pinto D , Chandra A , Crothers BA , Kurtycz DFI , Schmitt F . The international system for reporting serous fluid cytopathology—diagnostic categories and clinical management. J Am Soc Cytopathol. 2020;9(6):469–477. 32620534 10.1016/j.jasc.2020.05.015

[20] Potdar S , Ianevski A , Mpindi J‐P , Bychkov D , Fiere C , Ianevski P , et al. Breeze: an integrated quality control and data analysis application for high‐throughput drug screening. Bioinformatics. 2020;36(11):3602–3604. 32119072 10.1093/bioinformatics/btaa138 PMC7267830

[21] Yadav B , Pemovska T , Szwajda A , Kulesskiy E , Kontro M , Karjalainen R , et al. Quantitative scoring of differential drug sensitivity for individually optimized anticancer therapies. Sci Rep. 2014;4(1):5193. 24898935 10.1038/srep05193 PMC4046135

[22] Thompson JC , Fan R , Black T , Yu GH , Savitch SL , Chien A , et al. Measurement and immunophenotyping of pleural fluid EpCAM‐positive cells and clusters for the management of non‐small cell lung cancer patients. Lung Cancer. 2019;127:25–33. 30642547 10.1016/j.lungcan.2018.11.020 PMC6657687

[23] Hirte HW , Clark DA , Mazurka J , O'Connell G , Rusthoven J . A rapid and simple method for the purification of tumor cells from ascitic fluid of ovarian carcinoma. Gynecol Oncol. 1992;44(3):223–226. 1531803 10.1016/0090-8258(92)90046-l

[24] Dhupar R , Okusanya OT , Eisenberg SH , Monaco SE , Ruffin AT , Liu D , et al. Characteristics of malignant pleural effusion resident CD8+ T cells from a heterogeneous collection of tumors. Int J Mol Sci. 2020;21(17):6178. 32867034 10.3390/ijms21176178 PMC7503595

[25] Salaroglio IC , Kopecka J , Napoli F , Pradotto M , Maletta F , Costardi L , et al. Potential diagnostic and prognostic role of microenvironment in malignant pleural mesothelioma. J Thorac Oncol. 2019;14(8):1458–1471. 31078776 10.1016/j.jtho.2019.03.029

[26] Ye N , Cai J , Dong Y , Chen H , Bo Z , Zhao X , et al. A multi‐omic approach reveals utility of CD45 expression in prognosis and novel target discovery. Front Genet. 2022;13:928328. 36061172 10.3389/fgene.2022.928328 PMC9428580

[27] Scharpenseel H , Hanssen A , Loges S , Mohme M , Bernreuther C , Peine S , et al. EGFR and HER3 expression in circulating tumor cells and tumor tissue from non‐small cell lung cancer patients. Sci Rep. 2019;9(1):7406. 31092882 10.1038/s41598-019-43678-6 PMC6520391

[28] Loh C‐Y , Chai JY , Tang TF , Wong WF , Sethi G , Shanmugam MK , et al. The E‐cadherin and N‐cadherin switch in epithelial‐to‐mesenchymal transition: signaling, therapeutic implications, and challenges. Cells. 2019;8(10):1118. 31547193 10.3390/cells8101118 PMC6830116

[29] Gupta PB , Pastushenko I , Skibinski A , Blanpain C , Kuperwasser C . Phenotypic plasticity: driver of cancer initiation, progression, and therapy resistance. Cell Stem Cell. 2019;24(1):65–78. 30554963 10.1016/j.stem.2018.11.011 PMC7297507

[30] Deshmukh S , Saini S . Phenotypic heterogeneity in tumor progression, and its possible role in the onset of cancer. Front Genet. 2020;11:604528. 33329751 10.3389/fgene.2020.604528 PMC7734151

[31] Kitayama J , Emoto S , Yamaguchi H , Ishigami H , Watanabe T . CD90(+) mesothelial‐like cells in peritoneal fluid promote peritoneal metastasis by forming a tumor permissive microenvironment. PLoS One. 2014;9(1):e86516. 24466130 10.1371/journal.pone.0086516 PMC3897715

[32] Nakamura IT , Ikegami M , Hasegawa N , Hayashi T , Ueno T , Kawazu M , et al. Development of an optimal protocol for molecular profiling of tumor cells in pleural effusions at single‐cell level. Cancer Sci. 2021;112(5):2006–2019. 33484069 10.1111/cas.14821 PMC8088920

[33] Vona G , Sabile A , Louha M , Sitruk V , Romana S , Schütze K , et al. Isolation by size of epithelial tumor cells: a new method for the Immunomorphological and molecular characterization of circulating tumor cells. Am J Pathol. 2000;156(1):57–63. 10623654 10.1016/S0002-9440(10)64706-2 PMC1868645

[34] Basak SK , Veena MS , Oh S , Huang G , Srivatsan E , Huang M , et al. The malignant pleural effusion as a model to investigate intratumoral heterogeneity in lung cancer. PLoS One. 2009;4(6):e5884. 19536353 10.1371/journal.pone.0005884 PMC2697051

[35] Saini VM , Oner E , Ward M , Hurley S , Henderson BD , Lewis F , et al. A comparative study of circulating tumor cell isolation and enumeration technologies in lung cancer. *bioRxiv*. 2024 10.1101/2024.02.05.578972 PMC12234383 39105395

[36] Drucker A , Teh EM , Kostyleva R , Rayson D , Douglas S , Pinto DM . Comparative performance of different methods for circulating tumor cell enrichment in metastatic breast cancer patients. PLoS One. 2020;15(8):e0237308. 32790691 10.1371/journal.pone.0237308 PMC7425969

[37] Gorges TM , Tinhofer I , Drosch M , Röse L , Zollner TM , Krahn T , et al. Circulating tumour cells escape from EpCAM‐based detection due to epithelial‐to‐mesenchymal transition. BMC Cancer. 2012;12:178. 22591372 10.1186/1471-2407-12-178 PMC3502112

[38] Akhter MZ , Sharawat SK , Kumar V , Kochat V , Equbal Z , Ramakrishnan M , et al. Aggressive serous epithelial ovarian cancer is potentially propagated by EpCAM+CD45+ phenotype. Oncogene. 2018;37(16):2089–2103. 29379166 10.1038/s41388-017-0106-y

[39] Patriarca C , Macchi RM , Marschner AK , Mellstedt H . Epithelial cell adhesion molecule expression (CD326) in cancer: a short review. Cancer Treat Rev. 2012;38(1):68–75. 21576002 10.1016/j.ctrv.2011.04.002

[40] de Wit S , van Dalum G , Lenferink ATM , Tibbe AGJ , Hiltermann TJN , Groen HJM , et al. The detection of EpCAM(+) and EpCAM(−) circulating tumor cells. Sci Rep. 2015;5:12270. 26184843 10.1038/srep12270 PMC4505332

[41] Antonangelo L , Sales RK , Corá AP , Acencio MMP , Teixeira LR , Vargas FS . Pleural fluid tumour markers in malignant pleural effusion with inconclusive cytologic results. Curr Oncol. 2015;22(5):e336–e341. 26628873 10.3747/co.22.2563 PMC4608406

[42] Aleksiev V , Markov D , Bechev K . Tumor markers in pleural fluid: a comprehensive study on diagnostic accuracy. Diagnostics. 2025;15(2):204. 39857088 10.3390/diagnostics15020204 PMC11765104

[43] Robak A , Kistowski M , Wojtas G , Perzanowska A , Targowski T , Michalak A , et al. Diagnosing pleural effusions using mass spectrometry‐based multiplexed targeted proteomics quantitating mid‐ to high‐abundance markers of cancer, infection/inflammation and tuberculosis. Sci Rep. 2022;12(1):3054. 35197508 10.1038/s41598-022-06924-y PMC8866415

[44] Dong T , Liang Y , Chen H , Li Y , Li Z , Gao X . Quantitative proteomics revealed protein biomarkers to distinguish malignant pleural effusion from benign pleural effusion. J Proteomics. 2024;302:105201. 38768894 10.1016/j.jprot.2024.105201

[45] Grigoriadou GΙ , Esagian SM , Ryu HS , Nikas IP . Molecular profiling of malignant pleural effusions with next generation sequencing (NGS): evidence that supports its role in cancer management. J Pers Med. 2020;10(4):206. 33139621 10.3390/jpm10040206 PMC7712846

[46] Sun Z , Li P , Wu Z , Li B , Li W , Zhao M , et al. Circulating CD45+EpCAM+ cells as a diagnostic marker for early‐stage primary lung cancer. Front Med Technol. 2022;4:982308. 36147748 10.3389/fmedt.2022.982308 PMC9487715

[47] Ishizawa K , Yamanaka M , Saiki Y , Miyauchi E , Fukushige S , Akaishi T , et al. CD45+CD326+ cells are predictive of poor prognosis in non‐small cell lung cancer patients. Clin Cancer Res. 2019;25(22):6756–6763. 31383733 10.1158/1078-0432.CCR-19-0545

[48] Gast CE , Silk AD , Zarour L , Riegler L , Burkhart JG , Gustafson KT , et al. Cell fusion potentiates tumor heterogeneity and reveals circulating hybrid cells that correlate with stage and survival. Sci Adv. 2018;4(9):eaat7828. 30214939 10.1126/sciadv.aat7828 PMC6135550

[49] Huang Z‐Y , Shao M‐M , Zhang J‐C , Yi F‐S , Du J , Zhou Q , et al. Single‐cell analysis of diverse immune phenotypes in malignant pleural effusion. Nat Commun. 2021;12(1):6690. 34795282 10.1038/s41467-021-27026-9 PMC8602344

