# Supplementary material for: Evaluation and modification of tumor cell isolation techniques from malignant effusions for rapid drug sensitivity testing
Source: Mol Oncol. 2025 Jun 17;19(9):2474–90. doi: 10.1002/1878-0261.70072 (PMC12420342; doi:10.1002/1878-0261.70072)
Supplement: Supplementary file 1 — Fig. S1. Anti‐EpCAM magnetic beads don't block the binding of anti‐EpCAM antibodies to their target epitopes. [file MOL2-19-2474-s002.docx]

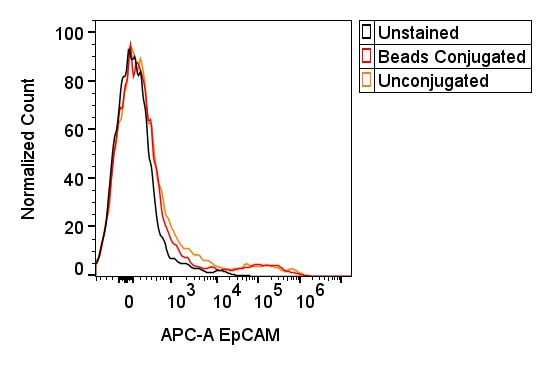


**Figure S1. anti EpCAM magnetic beads don't block the binding of anti-EpCAM antibodies to their target epitopes**. Representative EpCAM FACS histograms showing no significant change in fluorescence intensity of MPE cells following incubation with anti-EpCAM magnetic beads (“Beads conjugated” versus “Unconjugated”). The data is representative of three different samples of MPEs that were tested.
