# Supplementary material for: Evaluation and modification of tumor cell isolation techniques from malignant effusions for rapid drug sensitivity testing
Source: Mol Oncol. 2025 Jun 17;19(9):2474–90. doi: 10.1002/1878-0261.70072 (PMC12420342; doi:10.1002/1878-0261.70072)
Supplement: Supplementary file 2 — Table S1. Patients' clinical characteristics and %EpCAM+ in their MPE cells. [file MOL2-19-2474-s001.docx]

Table S1. Patients’ clinical characteristics and %EpCAM^+^ in their MPE cells.

| **ID** | **Age** | **Gender** | **Diagnosis** | **Cytology** | **%EpCAM^+^** |
| --- | --- | --- | --- | --- | --- |
| **1** | 68 | Male | CML | Neg | 0 |
| **2** | 73 | Male | Sarcoma | Neg | 0 |
| **3** | 81 | Female | Ovarian Carcinoma | Pos | 11.7 |
| **4** | 75 | Male | Lung Adenocarcinoma | Pos | 2.2 |
| **5** | 55 | Female | Lung Adenocarcinoma | Pos | 3.5 |
| **6** | 70 | Female | Lung Adenocarcinoma | Pos | 0.8 |
| **7** | 89 | Female | Meig's syndrome | Neg | 0 |
| **8** | 70 | Male | Lung Adenocarcinoma | Pos | 20 |
| **9** | 71 | Female | Lung Adenocarcinoma | Pos | 1.3 |
| **10** | 75 | Female | CHF | Neg | 0 |
| **11** | 55 | Female | Lung Adenocarcinoma | Pos | 2 |
| **12** | 55 | Female | Lung Adenocarcinoma | Pos | 1.5 |
| **13** | 78 | Female | Liver Cirrhosis | Neg | 0 |
| **14** | 86 | Female | Lung Adenocarcinoma | Neg | 40 |
| **15** | 66 | Male | Dialysis | Neg | 0 |
| **16** | 70 | Female | Lung Adenocarcinoma | Pos | 0.5 |
| **17** | 58 | Female | Ovarian Carcinoma | Pos | 20 |
| **18** | 64 | Male | Lung Adenocarcinoma | Pos | 50 |
| **19** | 68 | Female | Lung Adenocarcinoma | Pos | 6 |
| **20** | 88 | Male | CRF, CHF, Dialysis | Neg | 0 |
| **21** | 58 | Female | Ovarian Carcinoma | Pos | 50 |
| **22** | 88 | Female | Breast Adenocarcinoma | Pos | 3 |
| **23** | 88 | Female | Breast Adenocarcinoma | Pos | 4.5 |
| **24** | 44 | Female | Colorectal Carcinoma | Neg | 0 |
| **25** | 70 | Female | Ovarian Carcinoma | Neg | 0 |
| **26** | 88 | Female | Breast Adenocarcinoma | Pos | 5 |
| **27** | 85 | Female | Lung Adenocarcinoma | Pos | 1 |
| **28** | 82 | Female | Lung Adenocarcinoma | Pos | 0.5 |
| **29** | 67 | Female | Breast Adenocarcinoma | Pos | 61 |
| **30** | 69 | Female | Lung Adenocarcinoma | Pos | 80 |
| **31** | 57 | Male | Lung Adenocarcinoma | Pos | 0.3 |
| **32** | 74 | Female | Lung Adenocarcinoma | Neg | 0 |
| **33** | 73 | Female | Lung Adenocarcinoma | Neg | 0 |
| **34** | 72 | Male | Lung Adenocarcinoma | Neg | 0 |
| **35** | 53 | Female | Lung Adenocarcinoma | Pos | 30 |
| **36** | 62 | Female | Ovarian Carcinoma | Pos | 5.5 |
| **37** | 80 | Male | Lung Adenocarcinoma | Pos | 6 |
| **38** | 77 | Male | Lung Adenocarcinoma | Pos | 2.8 |
| **39** | 71 | Female | Lung Adenocarcinoma | Pos | 11.5 |
| **40** | 51 | Female | Lung Adenocarcinoma | Pos | 70 |
| **41** | 66 | Female | Ovarian Carcinoma | Pos | 70 |
| **42** | 76 | Male | Lung Adenocarcinoma | Pos | 8.9 |
| **43** | 67 | Female | Lung Adenocarcinoma | Pos | 2.5 |
| **44** | 71 | Female | Lung Adenocarcinoma | Pos | 5.5 |
| **45** | 72 | Female | Small cell carcinoma | Pos | 2 |
| **46** | 74 | Female | Ovarian Carcinoma | Pos | 2 |
| **47** | 61 | Female | Lung Adenocarcinoma | Pos | 84 |
| **48** | 39 | Female | Lung Adenocarcinoma | Pos | 40 |
| **49** | 70 | Male | Lung Adenocarcinoma | Pos | 14 |
| **50** | 74 | Female | Lung Adenocarcinoma | Pos | 56 |
| **51** | 75 | Female | Lung Adenocarcinoma | Pos | 8 |
| **52** | 60 | Female | Ovarian Carcinoma | Pos | 6 |
| **53** | 38 | Female | Breast Adenocarcinoma | Pos | 24 |
| **54** | 79 | Male | Lung Adenocarcinoma | Pos | 5.4 |
| **55** | 44 | Female | Lung Adenocarcinoma | Pos | 3 |
| **56** | 58 | Female | Lung Adenocarcinoma | Pos | 30 |
| **57** | 56 | Female | Lung Adenocarcinoma | Pos | 18 |
| **58** | 79 | Male | Lung Adenocarcinoma | Pos | 27 |
| **59** | 75 | Female | Lung Adenocarcinoma | Pos | 58 |
| **60** | 66 | Female | Lung Adenocarcinoma | Pos | 2 |
